# The moderating effect of care time on care-related characteristics and caregiver burden: differences between formal and informal caregivers of dependent older adults

**DOI:** 10.3389/fpubh.2024.1354263

**Published:** 2024-04-04

**Authors:** Eunmi Oh, SeolHwa Moon, Daum Chung, Rina Choi, Gwi-Ryung Son Hong

**Affiliations:** ^1^Research Institute of Nursing Science, Hanyang University, Seoul, Republic of Korea; ^2^Department of Nursing, Hoseo University, Cheonan-si, Republic of Korea; ^3^College of Nursing, Hanyang University, Seoul, Republic of Korea

**Keywords:** older adults, caregiver burden, formal caregivers, informal caregivers, care time, care attitude, moderating effect

## Abstract

**Objective:**

This study examined differences in care burden between formal and informal caregivers of dependent older adults according to care-related characteristics, and whether care time had a moderating effect on the relationship between care-related characteristics and caregiver burden.

**Methods:**

Participants were formal (*n* = 520) and informal caregivers (*n* = 142) of dependent older adults in South Korea. Caregiver burden was measured using the Korean version of the Zarit Burden Interview. Data were analyzed using hierarchical regression with interaction terms and moderation analysis.

**Results:**

Caregiver burden was higher for informal caregivers than formal caregivers. Factors associated with an increased risk of caregiver burden in both formal and informal caregiver of dependent older adults were caregivers’ stress, physical strain, and care time. Care time significantly moderated the relationship between care attitude and care burden only among formal caregivers. When formal caregivers’ care time was 1 standard deviation higher than the mean value, care attitude was significantly associated with care burden (b_simple_ = −0.903, SE = 0.106, *p* < 0.001).

**Conclusion:**

The caregiver burden of dependent older adults can be reduced by providing interventions to attenuate the effects of modifiable risk factors that were identified in this study. And to weaken the relationship between care attitude and burden of formal caregivers who have long care hours, a positive social atmosphere for care should be provided in addition to education. To realize sustainable care, policy considerations that reflect the results of this study will help solve the problem of formal and informal caregiver burden of dependent older adults.

## 1 Introduction

The proportion of the global population that is older adult is increasing rapidly, and accordingly, the need for care for dependent older adults with reduced ability to perform activities of daily living (ADL) is increasing (1). There are two main types of care for older people who are aging and need help taking care of themselves. The first is formal care, which generally refers to paid care provided by medical institutions or medically trained individuals for those in need (2). The second type of care is informal care, which refers to unpaid care provided by family, close relatives, friends, and neighbors. Both formal and informal care involve a variety of tasks, but informal caregivers rarely receive adequate training for these tasks (2). Informal care remains the most common source of home care in the United States, with the use of informal home care by older adults with disabilities increasing from 2004 to 2016; nearly three-quarters of older adults with disabilities received informal home care in 2016. In Korea, family members account for the highest percentage of main caregivers, but the proportion of formal caregivers is increasing continuously (3).

Caregiver burden refers to the multifaceted strain that a caregiver experiences over time while caring for others (4, 5), and if high, can have negative consequences such as decreased quality of care, decreased caregiver quality of life, and poor physical and psychological health of the caregiver (5). Previous reviews reported various factors related to the care burden of formal (6) and informal care givers (7–10). In a systematic review study (10), factors related to care burden were classified into three categories: care-recipient-, caregiver-, and society-related factors. Care-recipient factors influencing care burden were physical disability (11–13), mental dependency (11), and behavioral and psychological symptoms of dementia (13). Caregiver factors were gender (11), age (13–15), satisfaction with care (16), caring time (10, 17, 18), duration of caregiving (11), care attitude (19), and stress (6, 11). Lastly, the utilization of community services (20) and perceived social support (11, 20) were social factors reported to affect caregiver burden of care.

In Korea, due to the low birth rate and rapid increase in the older adult population, the aged population ratio is expected to reach more than 40% in 2050 (21). With the increase in the aged population, social demand for care has increased. Korea is based culturally on Confucian familism (patriarchal tradition), founded on the rule of filial piety (ideology) that believes that caring for parents is a duty. Because of this, families, mainly women, traditionally have provided older adults care within the home (22). In a recent survey, older adults who are receiving informal care by family members living with them, especially children and spouses, still account for 74.5% of those in need of care (23). However, due to changes in social situations such as changes in family structure (smaller families), women’s labor force participation, and an increase in the number of older adults living alone, care by children is expected to decrease in the future (24). Long-term informal care is a tremendous burden for informal caregivers (11), both older adults and caregivers of their family caregivers were found to be at increased risk of suicide based on a systematic literature review in Korea (9).

Under such a situation, to reduce the functional decline or gap in older adult care provided by families and the burden on informal caregivers, the long-term care insurance system was introduced as a public care system in 2008. As part of this system, the government developed and systematized jobs for formal caregivers to provide national long-term care benefits to older adults and their families (25). However, formal caregivers endure poor working conditions, low wages, unstable employment, high job intensity, and stress. In addition, formal care jobs are considered non-professional, non-respected jobs for women (25). In particular, the burden on formal caregivers in long-term care facilities (LTCFs) is remarkably high and is becoming a social problem in Korea (26). Despite this expansion of care infrastructure and an increase in the number of formal caregivers, criticism has been raised regarding poor quality in care services (27). In addition, rapid aging is considered a major cause of increasing national economic burden, and ensuring financial sustainability while providing stable older adult care is an important policy task for Korea.

Among the various factors identified in previous studies, care-related characteristics of caregivers are consistently the most important contributor to the care burden of formal and informal caregivers (6, 10). According to a national representative cross-sectional study in United States, the characteristics of the caregiver and the provision of care work were stronger determinants of caregiver burden than the characteristics of the care-recipients (28). Especially longer caring hours and the physically strenuous nature of formal and informal caregiver work increased in the past few years due to COVID-19, with a concomitant increase in psychosocial burden (29–31). The World Health Organization (WHO) emphasized the importance of continuity of care for providing integrated people-centered health services (32). Previous studies have suggested that an increase in caregiver burden has a negative effect on the continuity of care (6, 10). Existing studies on caregiver burden have focused primarily on family caregivers, with fewer studies of formal caregivers.

Recently, although many studies have been conducted on the caregiver burden of older adults in Korea, they separately have analyzed formal caregivers (26) or informal caregivers (9, 17). There were limitations in exploring and comparing the differences in the degree of care burden and influencing factors between formal and informal caregivers. Therefore, in this time of increasing caregiver diversity, we performed this study to examine differences in care burden between formal and informal caregivers of dependent older adults in terms of care-related characteristics and explored the moderating effect of daily care time on the relationship between caregivers’ care-related characteristics and caregiver burden. In Korea, culturally, the family functions as the basic system of society, and female-centered family care has been common for older adults. However, with the accelerating aging and diversification of society, the national need for formal care is being emphasized. Therefore, in Korea, reducing the burden of formal and informal care is an important policy task that can support stable care for older adults and ensure the sustainability of national finances. Based on this, we suggest practical measures to reduce caregiver burden and secure the continuity and quality of care services. This study will provide cultural, familial, and economic context that can provide new insight into older adult care in Korea.

## 2 Materials and methods

### 2.1 Study design

This study was a descriptive research study. Data analyzed for the current study were selected from a survey on caring status for dependent older adults and people with severe disabilities (PI, *corresponding author), that was conducted from August 2021 to October 2022. This study involved a total of 1,375 participants composed of 1,174 caregivers (665 caregivers for the dependent older adults, 509 caregivers for the disabled), and 201 care-recipients. To access formal and informal caregivers, we reached out to several healthcare facilities and centers that provide care services to older adults. Our selection criteria for these healthcare facilities were based on (1) location and accessibility, (2) proportion of older adults (≥ 65 years) residing in LTCFs (e.g., nursing homes, geriatric hospitals) versus community settings, and (3) proportion of primary caregivers in each setting. At the start of the survey, we aimed to recruit facilities and centers in Gyeonggi-do, Incheon, Seoul, Chungcheong-do, Jeolla-do, and Gyeongsang-do in proportion to the number of older adults in each area. However, due to challenges in recruiting facilities during the COVID-19 pandemic, we reached out to healthcare facilities located in Gyeonggi-do, Seoul, Chungcheong-do, and Jeolla-do. As a result, total participants were recruited from eight nursing homes, six geriatric hospitals, 20 community home visit centers, seven facilities for disabled people, 17 community-welfare centers, and seven other facilities in Seoul, Gyeonggi, Chungcheong, and Jeolla provinces in Korea.

The sample size for multiple regression analysis was assessed using G*Power 3.1 software (33) with a significance level (α) of 0.05, power (1- β) of 80%, medium effect size (f^2^) of 0.15, and 13 predictive factors. The minimum sample size was 131. In this study, data from 662 caregivers (520 formal caregivers, 142 informal caregivers) were used in the final analysis, excluding three incomplete responses (missing data for the Korean version of the Zarit Burden Interview) from a total of 665 participants.

### 2.2 Participants

The inclusion criteria uniformly applied to both formal and informal caregivers were as follows: (1) taking care of an adult aged 65 or older who was dependent in ADLs (graded 1–4 based on the Korean long-term care grade or not yet graded but difficulty in daily life with decreased mobility) (34) and (2) had been providing care for more than 1 month.

#### 2.2.1 Formal caregivers

In this study, formal caregivers were identified as individuals offering direct paid care services to older adults across diverse settings including homes, community settings, and institutions (2). This category encompassed care workers, unlicensed assistive personnel, and personal assistant services for dependent older adults in Korea.

#### 2.2.2 Informal caregivers

In this study, informal caregivers were defined as family members (spouses, children, siblings), friends, or neighbors who provide care to older adults without payment (2).

### 2.3 Data collection

The initial research plan involved in-person data collection from all caregivers. However, due to the COVID-19 pandemic, this became unrealistic. Consequently, strategic participant recruitment and data collection were carried out based on caregiver types and settings. Face-to-face and online surveys (through Google forms) were conducted concurrently. Of the total 662 participants, 322 (48.6%) participated in the online survey, while 340 (51.4%) took part in the research through face-to-face interactions.

#### 2.3.1 Formal caregivers

Formal caregivers working in facilities or centers underwent a systematic data collection process. Initially, contact was made with each facility and center, involving the distribution of official documents and research protocols. Upon approval, an individual, such as nurse, social worker, case manager, or director, was assigned to each facility for data collection. These designated individuals received training using a data collection manual and assisted other caregivers in the same facility to ensure a smooth response to the survey. Subsequently, research recruitment notices were posted, and for those who agreed to participate, data collection took place through printed or online surveys. The online survey link was shared with the personnel managing data collection within the facility for accessibility. All caregivers in the facilities were able to contact the principal investigator and researchers by phone for any clarifications. An optional step included communication with facility personnel to verify the reliability and accuracy of data on specific aspects as needed.

#### 2.3.2 Informal caregivers

For informal caregivers, the following process was employed. Initial contact with home care centers, public health centers, welfare agencies, and public hospitals was made to assess the possibility of coordinating with families of older adults. Upon obtaining consent, either home visits or center meetings were scheduled for one-on-one interviews with researchers or research assistants. For informal caregivers who preferred remote engagement, an online survey was administered as a Google form distributed via text message or email. Similar to the process with formal caregivers, a review of the entered data was conducted; if additional clarification was needed, informal caregivers were contacted for verification.

### 2.4 Measurements

#### 2.4.1 Care-recipient factors (adjustment variables)

Cognitive impairment was considered present if dementia was the main diagnosis or comorbidity of dependent older adults, and absent if no diagnosis of dementia was present. Activities of daily living (ADLs) of older adults were assessed using the Korean version of the Barthel Activities of Daily Living index (ADL index) (35), a standardized version of the Barthel ADL index in Korean (36). The Barthel ADL index consists of a total of 10 items: bowel control, bladder control, toilet use, grooming, eating, dressing, transfer, mobility, climbing stairs, and bathing. Each item has a score ranging from 0 (total dependence) to 1 (total independence), 0 (total dependence) to 2 (total independence) or 0 (total dependence) to 3 (total independence), with the total score ranging from 0 to 20. The higher the score, the more independent the care recipient is in daily living activities. Cronbach’s alpha of the Korean version of the Barthel ADL index was 0.92 in this study.

#### 2.4.2 Caregiver factors

Formal caregivers were defined as those who provided paid care (e.g., paid health-care assistants, long-term care workers, etc.) while informal caregivers referred to caregivers who provided unpaid care (e.g., family members, relatives, etc.). Gender and age of caregivers were included as adjustment variables.

Care-related characteristics of caregivers included relationship satisfaction, perceived stress, care attitude, and physical strain. Relationship satisfaction was measured by asking the question, “How satisfied are you with the mutual relationship you have with the older adult who is receiving care?” The score ranged from 1 (very dissatisfied) to 10 (very satisfied), and the higher the score, the higher the relationship satisfaction.

Perceived stress in daily life was measured using the Korean Version of the Perceived Stress Scale-10 (KPSS-10) (37), which is a standardized Korean version of PSS-10 (38). KPSS-10 consists of a total of 10 items, including negative responses (items 1, 2, 3, 6, 9, and 10) and positive responses (items 4, 5, 7, and 8). Each item is measured on a five-point Likert scale with a score ranging from 0 (not at all) to 4 (very frequent), with the total score ranging from 0 to 40. The higher the score, the higher the perceived stress. The reported Cronbach’s alpha at the time of development of the KPSS-10 was 0.75 (37) and it was calculated to be 0.74 in this study.

Care attitudes toward older adults (39) was used to assess caregivers’ care attitude, and were based on the Youth’s Attitudes Toward the Elderly (40) and the Maxwell-Sullivan Attitudes Scale Toward the Geriatric Patient (41). Care attitudes for older adults were assessed using a total of 17 items. Each item was measured on a 5-point Likert scale and had a score ranging from 1 (very negative) to 5 (very positive), with a total score range from 17 to 85. The higher the score, the more positive the care attitude. Cronbach’s alpha for this scale was 0.93 in this study.

Physical strain was measured by asking the question, “How do you rate the physical difficulty of the overall caring work you are currently doing?” The score ranged from 1 (not at all physically difficult) to 4 (very physically difficult); the higher the score, the higher the physical burden.

#### 2.4.3 Moderating factor

Daily care time was measured by asking the caregiver how many average caring hours they provided per day.

#### 2.4.4 Dependent factors

Caregiver burden was measured by the Korean version of ZBI (ZBI-K) (42); the reliability of this tool was verified in caregivers of care-recipients with dementia (43). ZBI-K has also been used to measure caregiver burden of caregivers caring for severely disabled adults and older adults (44, 45). ZBI-K consists of a total of 22 item. Each item is measured on a 5-point Likert scale and is scored from 0 (not at all) to 4 (almost always), with the total score ranging from 0 to 88; the higher the score, the greater the burden of caregiving. Cronbach’s alpha of ZBI-K was previously calculated to be 0.93 (42), and it was 0.93 in this study as well.

### 2.5 Statistical analysis

Characteristics of care-recipients and caregivers were summarized as frequencies. The significance of differences in the characteristics of formal and informal caregivers were analyzed by independent t-test and Chi-square test. Pearson’s correlation coefficients were used to identify bivariate relationships between the main variables. Hierarchical regression analysis was conducted to assess if daily care time had a moderating effect on the relationship between care-related characteristics (relationship satisfaction, perceived stress, care attitude, and physical strain) and care burden. Care-recipients’ cognitive impairment, ADLs, and caregivers’ gender and age were adjusted for in the first step, and four care-related variables (relationship satisfaction, perceived stress, care attitude, and physical strain) were entered into the regression model. In the second step, the moderating variable (care time) was entered into the model to investigate the effect of care time on care burden. In the third step, interaction terms between care-related characteristics (relationship satisfaction, perceived stress, care attitude, and physical strain) and care time were added into the model to confirm the moderating effect of care time on the relationship between care-related characteristics and care burden. All interaction term variables were standardized by centering the score at the mean in consideration of the multicollinearity problem that may occur when verifying interaction effects.

**Figure 1:**
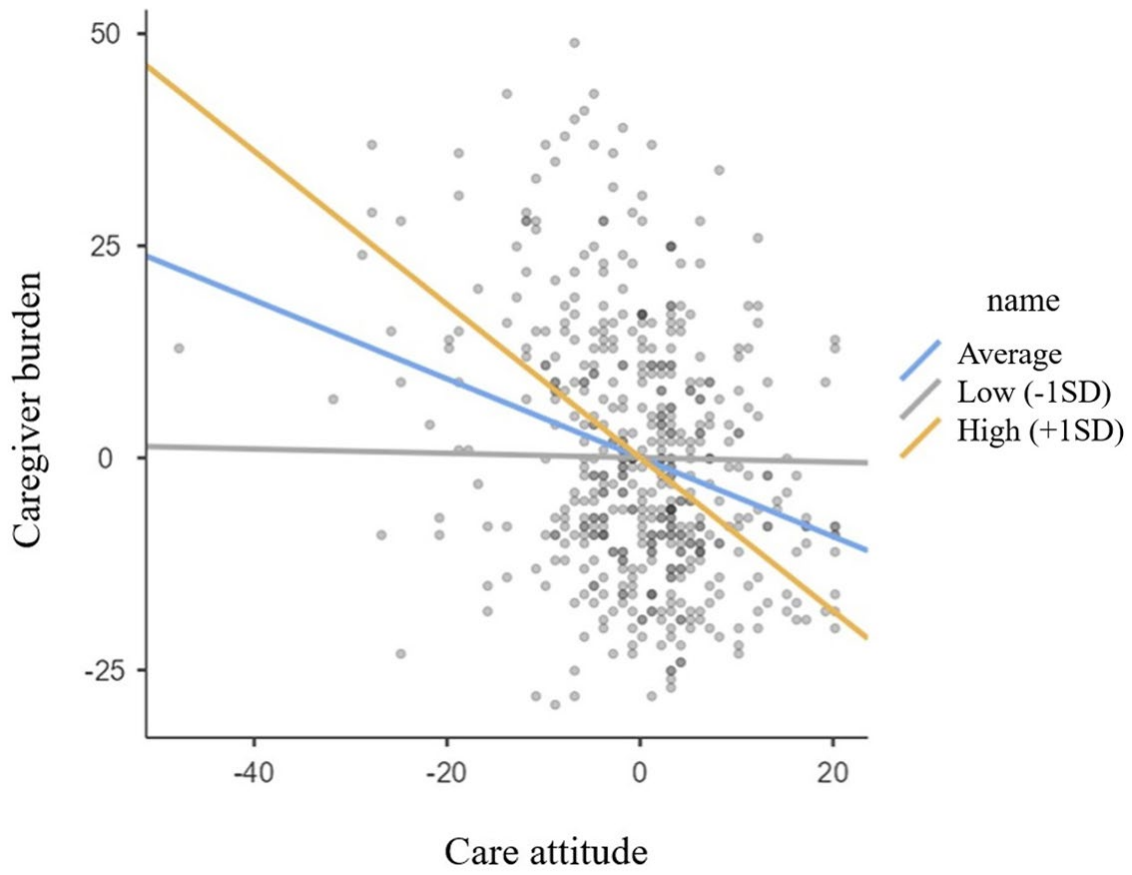
Simple slop plot for the moderation effect of daily care time on the relationship between care attitude and caregiver burden in formal caregivers.

Simple slope analysis was used to investigate significant interaction terms; the results of this analysis are presented in Figure 1. The relationship between care-related characteristics and caregiver burden was evaluated at three levels of the moderator, namely care time: low level (mean − 1 standard deviation, SD), average level (mean), and high level (mean + 1 SD) (46). All analyses were executed using IBM SPSS, version 23.0 (IBM Corp., Armonk, NY, United States) software and the Jamovi (47) medmod package (Tables 1–3).

**Table 1:** Characteristics of participants (*N* = 662).

Factors and variables	Total	Formal caregivers	Informal caregivers	*t* or *χ*^2^	*p*
	(*N* = 662)	(*n* = 520)	(*n* = 142)		
	*n* (%) or M ± SD	*n* (%) or M ± SD	*n* (%) or M ± SD		
Care-recipient factors					
Cognitive impairment				1.03	0.338
Yes	390 (58.9)	312 (60.0)	78 (54.9)		
No	272 (41.1)	208 (40.0)	64 (45.1)		
Activities of daily living	7.27 ± 6.00	6.64 ± 6.00	9.57 ± 5.25	−5.72	<0.001
Caregiver factors					
General characteristics					
Gender				0.68	0.404
Male	70 (10.6)	52 (10.0)	18 (12.7)		
Female	592 (89.4)	468 (90.0)	124 (87.3)		
Age (years)	61.15 ± 7.87	61.06 ± 6.93	61.49 ± 10.65	−0.46	0.645
Care-related characteristics					
Relationship satisfaction	7.45 ± 1.78	7.16 ± 1.74	7.49 ± 1.75	−2.01	0.817
Perceived stress	18.24 ± 4.77	17.41 ± 4.42	21.31 ± 4.80	−9.16	<0.001
Care attitude	64.25 ± 8.61	64.87 ± 8.75	58.74 ± 7.33	3.54	<0.001
Physical strain	3.91 ± 0.88	3.84 ± 0.89	4.14 ± 0.81	−2.21	<0.001
Moderating factor					
Care time (hours/day)	8.70 ± 6.77	8.59 ± 6.20	9.13 ± 8.57	−0.75	0.482
Dependent factor					
Caregiver burden	32.61 ± 16.33	29.17 ± 14.76	45.20 ± 15.61	−11.39	<0.001

**Table 2:** Correlations among the main variables (*N* = 662).

Variables		Formal caregiver (*n* = 520)					
		*r* (*p*)					
		1	2	3	4	5	6
1	Relationship satisfaction	1					
2	Stress	−0.150 (0.001)	1				
3	Care attitude	0.298 (0.001)	−0.342 (<0.001)	1			
4	Physical strain	−0.130 (0.003)	0.296 (<0.001)	−0.251 (<0.001)	1		
5	Care time	−0.059 (0.186)	−0.067 (0.127)	−0.172 (<0.001)	0.173 (<0.001)	1	
6	Caregiver burden	−0.184 (<0.001)	0.505 (<0.001)	−0.280 (<0.001)	0.463 (<0.001)	0.132 (<0.001)	1

Variables		Informal caregiver (*n* = 142)					
		*r* (*p*)					
		1	2	3	4	5	6
1	Relationship satisfaction	1					
2	Stress	−0.231 (0.006)	1				
3	Care attitude	0.232 (0.006)	−0.238 (0.004)	1			
4	Physical strain	−0.210 (0.014)	0.445 (<0.001)	−0.083 (0.327)	1		
5	Care time	0.048 (0.575)	0.117 (0.169)	0.122 (0.153)	0.225 (0.008)	1	
6	Caregiver burden	−0.241 (0.004)	0.624 (<0.001)	−0.227 (0.007)	0.496 (<0.001)	0.245 (0.003)	1

**Table 3:** Hierarchical regression analysis results (*N* = 662).

	Predictable variable	Formal caregiver (*n* = 520)														
		Step 1					Step 2					Step 3				
		*β*	SE	*t*	*p*	VIF	*β*	SE	*t*	*p*	VIF	*β*	SE	*t*	*p*	VIF
Model 1	Relationship satisfaction	−0.06	0.32	−1.67	0.096	1.11	−0.06	0.32	−1.74	0.083	1.11	−0.06	0.32	−1.68	0.093	1.14
	Stress	0.38	0.13	9.46	<0.001	1.24	0.40	0.13	10.00	<0.001	1.27	0.38	0.13	9.40	<0.001	1.33
	Care attitude	−0.05	0.07	−1.14	0.254	1.30	−0.02	0.07	−0.60	0.548	1.33	−0.04	0.07	−1.03	0.305	1.35
	Physical strain	0.34	0.82	8.79	<0.001	1.15	0.32	0.82	8.41	<0.001	1.17	0.32	0.81	8.48	<0.001	1.17
Model 2	Care time						0.14	0.10	3.53	<0.001	1.31	0.11	0.10	2.58	0.010	1.45
Model 3	Relationship satisfaction x Care time											0.03	0.06	0.67	0.504	1.37
	Stress x Care time											0.01	0.02	0.01	0.996	1.72
	Care attitude x Care time											−0.17	0.01	−3.82	<0.001	1.64
	Physical strain x Care time											−0.05	0.13	−1.13	0.259	1.32
	*R* ^2^	0.369					0.385					0.407				
	Adjusted *R*^2^	0.359					0.374					0.391				
	*F* (*p*)	36.30 (< 0.001)					34.39 (< 0.001)					25.89 (< 0.001)				
	*F* change (*p*)	12.34 (< 0.001)					12.43 (< 0.001)					4.56 (< 0.001)				

	Predictable variable	Informal caregiver (*n* = 142)														
		Step 1					Step 2					Step 3				
		*β*	SE	*t*	*p*	VIF	*β*	SE	*t*	*p*	VIF	*β*	SE	*t*	*p*	VIF
Model 1	Relationship satisfaction	−0.05	0.61	−0.82	0.416	1.14	−0.07	0.60	−1.04	0.300	1.15	−0.07	0.61	−1.03	0.303	1.15
	Stress	0.50	0.24	6.73	<0.001	1.39	0.48	0.24	6.56	<0.001	1.40	0.49	0.25	6.43	<0.001	1.44
	Care attitude	−0.10	0.15	−1.44	0.153	1.18	−0.11	0.15	−1.63	0.105	1.18	−0.10	0.15	−1.49	0.139	1.23
	Physical strain	0.24	1.38	3.22	0.002	1.41	0.22	1.38	2.93	0.004	1.44	0.21	1.42	2.73	0.007	1.49
Model 2	Care time						0.15	0.13	2.10	0.038	1.26	0.14	0.13	2.00	0.048	1.29
Model 3	Relationship satisfaction x Care time											−0.01	0.03	−0.10	0.922	1.77
	Stress x Care time											0.05	0.02	0.71	0.478	1.22
	Care attitude x Care time											−0.01	0.16	−0.16	0.876	1.40
	Physical strain x Care time											−0.02	0.07	−0.34	0.732	1.22
	*R* ^2^	0.498					0.515					0.518				
	Adjusted *R*^2^	0.466					0.480					0.466				
	*F* (*p*)	15.62 (< 0.001)					14.75 (< 0.001)					9.99 (<0.001)				
	*F* change (*p*)	29.82 (< 0.001)					4.41 (0.038)					0.17 (0.954)				

Adjustment variables: care-recipient factors: cognitive impairment, activities of daily living; caregiver factors-gender: age. *β*, standardized coefficients derived from the final step; *R*^2^, explanation rate.

## 3 Results

### 3.1 Descriptive statistics and preliminary analysis

Dependent older adults cared for by formal caregivers had more cognitive decline and were more dependent in ADLs than older adults cared for by informal caregivers.

Most caregivers were female (89.4%) and mean age was 61.15 (SD ± 7.87) years among all caregivers without a difference in gender (*p* = 0.404) or age (*p* = 0.645) between formal and informal caregivers. Relationship satisfaction had an average score of 7.45 ± 1.78 for all participants, with no significant difference between formal and informal caregivers (*p* = 0.817). Formal caregivers had lower perceived stress scores than informal caregivers (formal 17.41 ± 4.42 vs. informal 21.31 ± 4.80) and higher care attitude scores (formal 64.87 ± 8.75 vs. informal 58.74 ± 7.33). However, they reported less physical strain (formal 3.84 ± 0.89 vs. informal 4.14 ± 0.81; all *p* < 0.001).

Daily care time was 8.70 ± 6.77 h per day on average for all participants and was similar between formal and informal caregivers (*p* = 0.482). Caregiver burden of formal caregivers (29.17 ± 14.76) was significantly lower than that of informal caregivers (45.20 ± 15.61; *p* < 0.001).

### 3.2 Correlation analysis

According to Pearson’s correlation analyses, caregiver burden was significantly negatively associated with relationship satisfaction (*r* = −0.18, *p* < 0.001 vs. *r* = −0.24, *p* = 0.004) and care attitude (*r* = −0.28, *p* < 0.001 vs. *r* = −0.23, *p* = 0.007), and positively associated with perceived stress (*r* = 0.51 vs. *r* = 0.62, *p* < 0.001), physical strain (*r* = 0.46 vs. *r* = 0.50, *p* < 0.001), and care time (*r* = 0.13, *p* < 0.001 vs. *r* = 0.25, *p* = 0.003) in both formal and informal caregivers.

### 3.3 Hierarchical regression analysis

Four care-related characteristics, namely relationship satisfaction, perceived stress, care attitude, and physical strain were entered in the regression model for formal caregivers in step 1 and accounted for 35.9% of the variance in caregiver burden. Perceived stress (*β* = 0.38, *p* < 0.001) and physical strain (*β* = 0.34, *p* < 0.001) positively predicted caregiver burden (*F* = 36.30, *p* < 0.001).

Introduction of the moderating variable of care time in step 2 accounted for an additional 1.5% of the variance in caregiver burden (*β* = 0.14, *p* < 0.001), with 37.4% of the variance accounted for (F change = 12.43, *p* < 0.001). Perceived stress (*β* = 0.40, *p* < 0.001) and physical strain (*β* = 0.32, *p* < 0.001) still positively predicted caregiver burden (*F* = 34.39, *p* < 0.001).

In step 3, when the four interaction terms were introduced, only the interaction between care attitude and care time accounted for an additional component of the variance (1.7% of the variance in caregiver burden, *β* = −0.17, *p* < 0.001, F change = 4.56, *p* < 0.001). Even after adding the interaction terms into the regression model, perceived stress (*β* = 0.38, *p* < 0.001), physical strain (*β* = 0.32, p < 0.001), and care time (*β* = 0.11, *p* = 0.010) positively predicted caregiver burden with a total explained variance of 39.1% (*F* = 25.89, *p* < 0.001).

For informal caregivers, the four care-related characteristics accounted for 46.6% of the variance in caregiver burden (step 1). Perceived stress (*β* = 0.50, *p* < 0.001) and physical strain (*β* = 0.24, *p* = 0.002) positively predicted caregiver burden (*F* = 15.62, *p* < 0.001).

When care time was introduced in step 2, it explained an additional 1.4% of the variance in caregiver burden (*β* = 0.15, *p* = 0.038) (F change = 4.41, *p* = 0.038). Perceived stress (*β* = 0.48, *p* < 0.001) and physical strain (*β* = 0.22, *p* = 0.004) still positively predicted caregiver burden. The total explained variance was 48.0% (*F* = 14.75, *p* < 0.001).

Introduction of the four interaction terms in step 3 did not explain any additional variance in caregiver burden; rather, the total variance in caregiver burden decreased compared to step 2 (46.6%). Even after adding the interaction terms to the regression model, however, perceived stress (*β* = 0.49, *p* < 0.001), physical strain (*β* = 0.21, *p* = 0.007), and care time (*β* = 0.14, *p* = 0.048) still positively predicted caregiver burden (*F* = 9.99, *p* < 0.001).

### 3.4 Further analysis of the moderating effect of care time in formal caregivers

We further explored the moderating effect of care time using a simple slope analysis. Figure 1 depicts the moderating effect of care time on the relationship between care attitude and caregiver burden in formal caregivers. This analysis evaluated the relationship between care attitude and caregiver burden according to care time (low, average, or high). When care time was low, the relationship between care attitude and caregiver burden was negative, but not significant (b _simple_ = −0.026, SE = 0.106, *p* = 0.809). When care time level was average, this relationship was again negative, but significantly increased in magnitude (b _simple_ = −0.464, SE = 0.072, *p* < 0.001) and it further significantly increased in magnitude when care time was high (b _simple_ = −0.903, SE = 0.106, *p* < 0.001). The care time of formal caregivers moderated the relationship between care attitude and caregiver burden, and the effect of care attitude on care burden was also moderated differently according to the amount of care time. In other words, care attitude was most strongly associated with lower caregiver burden when the formal caregiver reported high levels of care time.

## 4 Discussion

We examined the differences in care burden between formal and informal caregivers of dependent older adults according to care-related characteristics, and analyzed whether daily care time has a moderating effect on the relationship between caregivers’ care-related characteristics and care burden. This study is, to the best of our knowledge, the first study to identify differences in these associations between formal and informal caregivers of dependent older adults.

Importantly, we confirmed that perceived stress, physical strain, and care time, in descending order, increased the care burden of formal and informal caregivers, indicating that the effects of care time on caregiver burden are lower than those of stress and physical strain. This finding is consistent with existing studies that have reported that perceived stress and physical burden affect caregiver burden (6, 48).

Our results are similar to those of a previous study that analyzed differences in caregiver burden and potential overload between formal and informal caregivers in Australia (4); that study reported eight subthemes related to caregiver burden. Both formal and informal caregivers reported psychological burden as the most serious burden, followed by physical burden (e.g., back pain), whereas limited time resources, such as limited private time, were the subtheme least related to care burden.

Caregiver burden is widely accepted as a stress-related concept in caregiving studies. In a recent conceptual analysis, care burden of formal caregivers of vulnerable older adults in nursing home was understood as a complex response to the physical, psychological, emotional, social, and financial stressors associated with the care experience (49). The authors defined the care burden of a nursing home’s formal caregiver as “the demands of caring for dependent older adults with a level of competency and responsibility within the context of perceived stress” (49). In a review study on the determinants of burden in informal caregivers, multidimensional determinants of objective and subjective care burden were explained based on the Adapted Stress Model (ASM) (11). General stressors such as caregiving duration and recipient’s functional status contributed to care burden, in addition to intrapsychic stressors such as role conflict and role overload due to restrictions on caregivers’ time (11).

More importantly, moderation analysis of formal caregivers confirmed a significant interaction between care attitude and care time on caregiver burden. Care time appears to moderate the relationship between care attitude and care burden in formal caregivers of older adults, with higher care burden related to longer caring time. This suggests that caregiver burden may be buffered by a positive care attitude when care time is longer. This is a new finding that for the first time establishes a direct link between caregiver burden and care time as a predictor of caregiver burden in both formal and informal caregivers. Furthermore, our findings indicate that care time also moderates the relationship between care attitude and caregiver burden in formal caregivers.

Previous studies have examined the relationship between care time and care burden (18, 50). Increased caregiver time was found to be associated with negative outcomes (51). In particular, as caregiving time increased, the physical and mental burden caused by the care activities increased, causing fatigue, and primary caregivers of stroke survivors who provide care for more than 6 h a day were 2.8 times more likely to be depressed than caregivers who spent less than 6 h a day caregiving (51).

During COVID-19 in Korea, interpersonal contact was minimized to prevent the spread of this disease, and preventive cohort isolation (all staff and care-recipients had to live and work only at the facility and were prohibited from having direct contact with the outside world) was implemented at facilities for older adults (e.g., LTCFs, geriatric hospitals). In addition, as most types of community care services provided to the older adults were discontinued, formal and informal caregivers’ working hours increased. These changes unsurprisingly increased caregiver burden (52, 53). Globally, caregivers complained of increased job stress, anxiety, and depression due to lack of manpower, overtime, and work overload during the COVID-19 pandemic (29–31, 54).

A positive attitude is part of the coping process that is the primary defense for protecting mental health in situations that are rated as stressful. During the COVID-19 pandemic, nurses’ positive attitudes improved quality of professional life, reduced burnout and secondary traumatic stress, and improved compassion satisfaction (55). In addition, healthcare professionals’ positive attitude was a strong protective factor against stress (56).

In psychology, attitude refers to an individual’s willingness to respond in a particular way to a person, situation, or idea as reflected in the cognitive, affective, and behavioral domains based on experience (57). Social psychologists have long been interested in understanding attitudes because of the belief that attitudes strongly influence behaviors, decisions, and judgments. Quality of care depends on caregivers’ attitudes toward caring for older adults in LTCFs (58). In a study to understand the impact of knowledge and attitude on the care quality of the caregivers of older adults residing in LTCFs in China, it was found that caregivers’ knowledge and attitudes affected their practice, and quality of care improved with an improvement in the knowledge and attitude of caregivers (58).

Therefore, based on the results of previous studies, formal caregivers in this study likely experienced increased caregiver burden due to physical strain, stress, and increased care time during the COVID-19 pandemic period, but the group with longer working hours used positive attitude as a means of coping, thereby reducing their caregiver burden.

Consistent with existing literature related to care time as an important contributor to caregiving burden (17, 18, 50), increased care time predicted an increase in the care burden of informal caregivers in this current study. Furthermore, 13 of 32 studies considered in a systematic review on care burden in families with dementia reported that care time had a significant negative relationship to family care burden (10). The current study was conducted during the COVID-19 pandemic (August 2021 to October 2022), and average care time per day was 8.70 h (formal caregivers 8.59 h, informal caregiver 9.13 h), compared to 7.50 h in Australia (4) and 7.34 h in Korea (17) in family caregivers before COVID-19.

Contrary to our findings, informal caregivers’ care time did not affect caregiving burden in community-dwelling older adults and family caregivers living in the United States based on a nationally representative survey (28), and a study from Brazil reported that even daily care times of more than 12 h did not affect caregiver burden. Since the level of care burden may vary depending on the age of the informal caregiver, differences in the ages of informal caregivers in this study and previous studies may have resulted in the discrepant findings; careful interpretation of our research results is therefore necessary. However, there is no dispute that the longer the care time, the greater the negative effects; as informal caregivers increase their care time, they tend to report poorer health status, are more often current smokers, less physically active, and more obese (59).

In Korea, although the Labor Standards Act limits the maximum working hours for formal caregivers to 52 h per week, actual working hours in reality are still longer than legal regulations, including shift time and handover time (53); the daily working hours of formal caregivers at LTCFs was reported to be 9.5 h/day (60). In addition, among informal caregivers in Korea, poor quality of life due to caregiving is particularly evident among women, and as care time increases, negative consequences such as reduced labor participation, increased likelihood of quitting, and decreased quality of life are increased (24). Therefore, the implication of this study in Korea is that the formal caregiver burden can be buffered if the group with high care time experiences a positive care attitude. Additionally, based on this study, we suggest that formal caregivers with long care hours make efforts to have a positive care attitude (e.g., by receiving education and creating a social atmosphere) to decrease the care burden on formal caregivers.

This study has some limitations. In terms of research design, we analyzed antecedent variables for care burden using cross-sectional analysis, thus we were unable to assess causal relationships, as longitudinal studies are required to determine cause and effect. Additionally, because our sample was based on convenience sampling, a more representative sample should be used to confirm our findings. Limitations related to the research method including measurement tools and survey questions are as follows. In previous studies, conflict relationships with care recipients were a major risk factor for stress among family caregivers of older adults (31), and caregiver burden differed depending on the relationship between the caregiver and the older adults (61). However, we did not examine differences in burden according to the relationship between informal caregivers and older adults; this should be done in future studies. Additionally, the caregiving satisfaction of informal caregivers caring for older adults who need full-time help on basic daily activities was shown to have a significant impact on the burden of care (16). In previous studies, caregiving satisfaction was evaluated with the Caregiver Satisfaction Assessment Index (CSAI), which consists of 30 statements about positive aspects of caregiving. However, in this study, only one question was used to evaluate relationship satisfaction; more comprehensive evaluations of caregiving satisfaction should be incorporated in future studies.

Having family members assisting with daily care would help ease the burden on those responsible for primary care. Therefore, future study on caregiver burden should include any types of supports including available secondary caregivers, perceived social support (18), and community services (20). Empirical studies have shown that caregivers who perform physical care-related tasks (bathing, using the toilet, etc.) are more likely to experience stress compared to caregivers who help with less personal tasks (cleaning, mowing the lawn, etc.) (62). However, as current study only measured the responses to overall physical care and not those specifically related to care tasks, future research about physical strain is suggested to measure and to reflect the types of tasks performed by caregivers.

In previous studies, disease type (10, 61) and trajectory (63) were reported to affect the caregiver burden. In particular, the caregiver burden is high for dementia (61), stroke (12), and schizophrenia (19). In cases of dementia, the caregiver burden differs depending on the type of dementia (Lewy body disease > Alzheimer’s disease) (61). Additionally, in dementia, the burden of care increased over time according to the disease trajectory (63). Therefore, future research is suggested to include variables for disease type and trajectory. Finally, the COVID-19 pandemic and associated containment measures likely functioned as situational stressors and may have affected our findings. Future research should be conducted to confirm our findings under common stressors and life situations.

Despite these limitations, this study provides evidence that stress, physical strain, and care time were risk factors for caregiver burden in both formal and informal caregivers, but that care time had a moderating effect between care attitude and caregiver burden only in formal caregivers. As the demand for formal and informal care gradually increases due to the rapid aging of the population, the caregiver burden is also increasing. The caregiver burden has negative consequences on caregivers and care recipients. Until now, economic growth based on efficiency has been the goal of Korean society. However, the caregiver burden accompanying a low birth rate and aging is becoming a social problem, and the sustainability of society is threatened by limited national finances. Therefore, to provide sustainable care, based on the results of this study, we propose provide interventions to reduce the effects of modifiable risk factors for care burden. This study also suggests that a policy is needed to expand formal and informal care support policies and differentially support care time depending on the characteristics of the caregiver. The results of this study will have a positive impact on reducing the burden of care on care providers, including informal care providers who provide mainly care in a patriarchal culture. In addition, from a financial perspective, the study could have a positive impact on the employment rate of informal family caregivers or on the effective use of the government’s formal care-related finances.

## Data availability statement

The dataset presented in this document is not immediately available due to informed consent of the research participants and did not include public data deposition. Requests for access to datasets should be directed to the corresponding author.

## Ethics statement

The studies involving humans were approved by Hanyang University Institutional Review Board. The studies were conducted in accordance with the local legislation and institutional requirements. The participants provided their written informed consent to participate in this study.

## Author contributions

EO: Conceptualization, Formal analysis, Methodology, Writing – original draft, Writing – review & editing. SM: Formal analysis, Writing – review & editing. DC: Data curation, Visualization, Writing – review & editing. RC: Visualization, Writing – review & editing. GR-H: Conceptualization, Funding acquisition, Investigation, Project administration, Supervision, Writing – review & editing.
